# Ciprofloxacin-Collagen Conjugate in the Wound Healing Treatment

**DOI:** 10.3390/jfb3020361

**Published:** 2012-05-15

**Authors:** Francesco Puoci, Cristiana Piangiolino, Francesco Givigliano, Ortensia Ilaria Parisi, Roberta Cassano, Sonia Trombino, Manuela Curcio, Francesca Iemma, Giuseppe Cirillo, Umile Gianfranco Spizzirri, Donatella Restuccia, Rita Muzzalupo, Nevio Picci

**Affiliations:** 1 Department of Pharmaceutical Sciences, University of Calabria, Edificio Polifunzionale, Arcavacata di Rende (CS) 87036, Italy; Email: ortensiailaria.parisi@unical.it (O.I.P.); roberta.cassano@unical.it (R.C.); sonia.trombino@unical.it (S.T.); manuela.curcio@unical.it (M.C.); francesca.iemma@unical.it (F.I.); giuseppe.cirillo@unical.it (G.C.); g.spizzirri@unical.it (U.G.S.) ; donatella.restuccia@unical.it (D.R.); rita.muzzalupo@unical.it (R.M.); nevio.picci@unical.it (N.P.);; 2 Principium Europe, Via Como 45, Solaro (MI) 20020, Italy; Email: c.piangiolino@principium-bsi.com; 3 Department of Thoracic Surgery, Policlinico Universitario Germaneto-Fondazione Tommaso Campanella, Campus Universitario “Salvatore Venuta” Viale Europa, Località Germaneto 88100, Italy; Email: Catanzaro francescogiv@gmail.com

**Keywords:** protein, ciprofloxacin, quinolones, biomedical application, antibiotic, antimicrobial, infection, wound healing, fibroblast proliferation

## Abstract

The synthesis of a novel functional biomaterial for wound healing treatment was carried out by adopting a free-radical grafting procedure in aqueous media. With this aim, ciprofloxacin (CFX) was covalently incorporated into collagen (T1C) chains employing an ascorbic acid/hydrogen peroxide redox pair as biocompatible initiator system. The covalent insertion of CFX in the polymeric chains was confirmed by FT-IR and UV analyses, while an antibacterial assay demonstrated the activity of the synthesized conjugate against *Staphylococcus* *aureus* and Escherichia *coli*, microorganisms that commonly infect wounds. A catechin blended conjugate was also tested in order to evaluate the ability to influence fibroblast cell growth. The observed antibacterial activity and stimulation of fibroblast growth support the applicability of CFX-T1C conjugate in wound treatment encouraging the healing process.

## 1. Introduction

Collagen is the most abundant protein in the human body [1,2], accounting for 30% of the total proteins. In normal tissues, collagen provides tensile strength and structural integrity. The structural complexity involves numerous types of collagen associated with tissue-speciﬁc expression, organization into heteropolymeric or homopolymeric ﬁbrils of a uniform size, unique post-translational processing and interaction with proteoglycans, adhesion glycoproteins, cells, and other collagens.

Collagen provides structural integrity for connective tissues, such as dermis, bone, cartilage, tendon, ligament, and internal organs. Due to the wide distribution throughout various tissues, collagen represents one of the most abundant naturally occurring proteins on earth and also one of the most important macromolecules for different biomedical applications: drug delivery as drug carrier, tissue engineering as polymeric scaffold, natural biodegradable sutures and wound dressing [3,4]. In the literature, several studies report on the application of collagen as a device for the release of antibiotic drugs [5], but the possibility to couple this protein with intrinsic antibacterial activity represents an important challenge. In this study, we report the development of an ecofriendly strategy to achieve a fluoroquinolone/collagen conjugate in which the drug is covalently bound to the protein.

Modern surgical practices have markedly reduced the rate of wound infections. However, no reduction in infection rates has occurred during the late 20th century, despite further developments in surgical techniques and antimicrobial prophylaxis. Surgical patients tend to be sicker and to undergo more complex operations. Surgical site infections (SSIs) have enormous clinical and ﬁnancial implications. Higher infection rates translate into higher morbidity and mortality, as well as higher costs to the hospital, patient, and society as a whole. 

**Figure 1:**
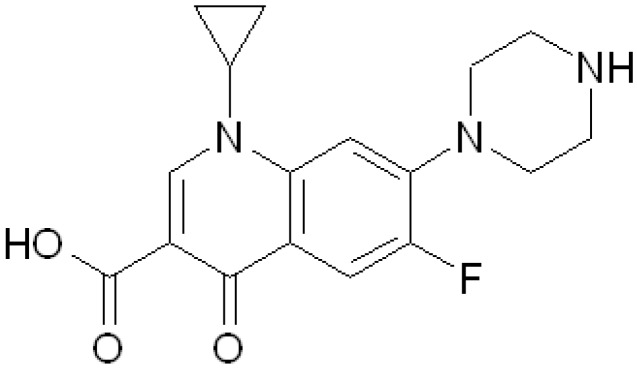
Chemical structure of Ciprofloxacin (CFX).

In this work the synthesis and of a Ciprofloxacin-Collagen conjugate (CFX-T1C) for wound healing applications was performed. Ciprofloxacin (CFX, Figure 1) is a synthetic broad-spectrum antibiotic belonging to the fluoroquinolone class, and it is effective against many gram-positive and gram-negative bacteria, including some strains resistant to other agents such as penicillins.

Although CFX is commonly administrated orally, several adverse effects have been recorded in treated patients [6]. CFX-T1C could be an alternative medical approach in wound healing, such as in diabetic foot. The local administration, indeed, achieves very high concentrations of the therapeutic agent at the site of action while the serum concentrations remain well below toxic levels.

The *in vitro* ability of CFX-T1C conjugate was also evaluated in the presence of a natural compound, such as catechin (CT), in order to stimulate ﬁbroblast growth.

Specifically, we answered to the following questions: Is it possible to prepare an antibiotic-collagen conjugate (CFX-T1C) in an ecofriendly and easy way? Does CFX-T1C show antibacterial activities? Can a CFX-T1C device (combining with catechin) successfully increase fibroblast proliferation?

## 2. Experimental Section

### 2.1. Materials

Collagen (from porcine tendon, Type I) (T1C), ciprofloxacin (CFX), (+)-catechin hydrate (CT), hydrogen peroxide (H_2_O_2_), ascorbic acid (AA), dipotassium hydrogen phosphate, potassium dihydrogen phosphate, calcium chloride, formaldehyde, tryptic soy agar (TSA), tryptic soy broth (TSB), agar, *Escherichia coli* (ATCC 25922), *Staphylococcus aureus *(ATCC 25923), acetic acid and sodium chloride were purchased from Sigma-Aldrich. 

NIH 3T3 Fibroblasts were purchased from American Type Culture Collection (ATCC).

Human foreskin ﬁbroblast (FS5) cells were cultured in Dulbecco's Modified Eagle Medium (DMEM, Sigma-Aldrich, United Kingdom), supplemented with 0.5% Fetal Bovine Serum (FBS). 

All used solvents were HPLC-grade and provided by Carlo Erba reagents (Milan, Italy).

### 2.2. Instruments

FT-IR spectra were recorded as KBr pellets on a Jasco FT-IR 4200. Dialysis membranes of 6-27/32'' Medicell International LTD (MWCO: 12–14000 Da) were employed. The freeze drier Micro Modulo, Edwards was employed. UV-Vis absorption spectra were obtained with a Jasco V-530 UV/Vis Spectrometer.

### 2.3. Synthesis of CFX-T1C Conjugate

The CFX-T1C conjugate was synthesized as follows: In a 50 mL glass ﬂask, 0.5 g of collagen were dissolved in 40 mL of a mixture of H_2_O/Acetic Acid (99/1, v/v), then 2.0 mL of H_2_O_2 _5.0 M containing 0.38 g of ascorbic acid were added and the mixture was maintained under stirring at 25 °C under atmospheric air. After 1 h, 0.2 g of CFX were added to the reaction mixture. After 6 h, the obtained polymer solution was introduced into dialysis tubes and dipped into a glass vessel containing distilled water at 20 °C for 48 h with eight changes of water. 

The resulting solution was frozen and dried with a freeze drier to afford a vaporous solid. 

The puriﬁed conjugate was checked to be free of unreacted antibiotic and any other compounds by UV spectral analysis after the puriﬁcation step.

### 2.4. Antibacterial Assay

*S. aureus* and *E. coli* were streaked out on TSA plates and incubated at 37 °C for 24 h [7]. A representative colony was lifted off with a wire loop and placed in 5 mL of TSB, which was then incubated with shaking at 37 °C for 24 h. At this stage, the cultures of *S. aureus* and *E. coli* contained approximately 10^9^ CFU/mL. Cultures of *S. aureus* and *E. coli* containing 10^7^ CFU/mL were prepared by dilution with TSB and were used for antibacterial tests.

The antibacterial activities of CFX-T1C were determined through the testing of different concentrations of the polymeric conjugate against *S. aureus* and *E. coli*. A range of concentrations (from 1,000 to 0.5 µg/mL) was prepared with sterile, double-deionized water (autoclaved) in 96-well microtiter plates. The test organisms (5 × 10^5^ CFU, 50 µL of TSB) were added to each well. In the end, each well contained 350 μL of water, 50 μL of TSB, and the test organism. The microtiter plates were incubated at 37 °C for 24 h in a shaker. At the end of this period, a small amount of the mixture from each well was pulled out and spread on agar plates with a swap, and the plates were incubated at 37 °C.

The growth of bacterial cells was observed on agar plates after 48 h incubation and the minimum inhibitory concentration (MIC), defined as the lowest concentration to inhibit visible growth, was determined. 

The antibacterial test was repeated at least four times employing water/TSB mixture (350 μL/50 μL) and water/TSB mixture (350 μL/50 μL) inoculated with each test bacterium in the microtiter plates as negative and positive controls, respectively. The growth of the test bacterium was observed in all wells with positive controls; on the contrary no growth was observed in the wells with the negative controls.

### 2.5. Cell Proliferation and Viability Assay

Human foreskin ﬁbroblast (FS5) cells were seeded at a density of 1 × 10^3^ cells per well in 96-well plates maintained at 37 °C in a humidiﬁed incubator of 5% CO_2_, 95% air atmosphere [8]. The medium was replaced after 48 h with 100 µL of Dulbecco’s Modified Eagle Medium (DMEM) containing 0.5% Fetal Bovine Serum (FBS). The CFX-T1C conjugate, doped with different concentrations of catechin, was initially dissolved in 1 mL of dimethyl sulfoxide (DMSO), ﬁltered to give the sterile stock solution and further diluted to give a ﬁnal concentration of 10 mg/mL of CFX-T1C in the wells. The CT concentration covers the range between 5–500 ng/mL. The ﬁnal volume of the medium was 200 µL per well. Two well plate columns were maintained on DMEM/0.5% FBS and DMEM/10% FBS as maintenance (negative) and positive growth stimulation controls, respectively. The cells were incubated for 48 h and cell growth determined using a neutral red uptake assay. After incubation the cells were washed with phosphate buffered saline (PBS), 100 µL freshly prepared neutral red solution was added to each well and the cells were incubated at 37 °C for 4 h. The neutral red was then removed by washing with 1% HCHO/1% CaCl_2_ and neutral red in the lysosomes was eluted with 100 µL of 1% acetic acid/50% ethanol over 30 min in an orbital shaker and the optical density measured at 540 nm using a 96-well plate reader. 

Three independent experiments were conducted and the results obtained are expressed as mean ± standard error of the mean of the absorbance. 

The data from the experiments were compared with the control (0.5% FBS) by one-way analysis of variance and Dunnet’s test. Differences at p < 0.05 were considered to be signiﬁcant.

## 3. Results and Discussion

### 3.1. Synthesis and Characterization of CFX-T1C Conjugate

The covalent insertion of ciprofloxacin in the collagen chains was performed by a free radical-induced grafting reaction. To this aim, a biocompatible and water-soluble system, ascorbic acid/hydrogen peroxide redox pair, was chosen as initiator system. 

The interaction mechanism between the two components of the redox pair involves the oxidation of ascorbic acid by H_2_O_2_ at room temperature with the formation of ascorbate and hydroxyl radical intermediates, which initiate the reaction [9,10,11,12]. 

**Figure 2:**
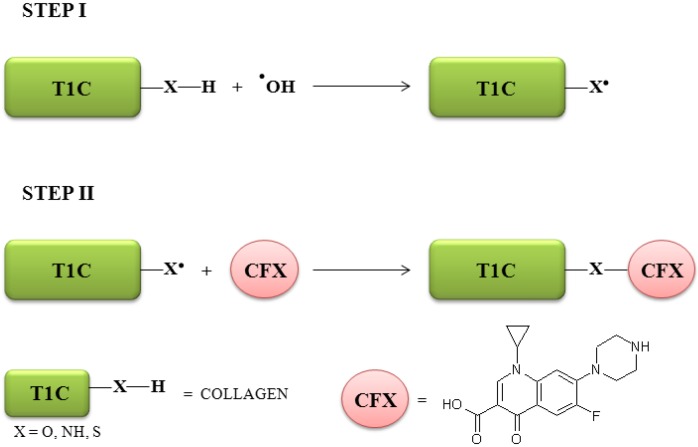
Insertion of CFX into collagen chain.

Compared to conventional initiator systems (*i.e.*, condense agents, esterification *etc*.) which require relatively high reaction temperatures to ensure rapid esterification, the grafting procedure shows several advantages. First of all, this kind of system does not generate toxic reaction products and allows the performance of the grafting process in aqueous media without any organic solvent; moreover, it is possible to perform the reaction at lower temperatures, reducing the risks of degradation. The best reaction conditions involve a first step designed for the collagen activation towards radical reactions in which hydroxyl radicals, generated by the interaction between redox pair components, attack the sensible residues in the side chains of protein producing radical species on the aminoacid structure, and a second step for the insertion of CFX onto the preformed T1C macroradicals (Figure 2).

The covalent incorporation of CFX into collagen chains was confirmed by performing FT-IR and UV-Vis analyses and the amount of CFX bound per gram of polymeric conjugate was 25 mg. 

**Figure 3:**
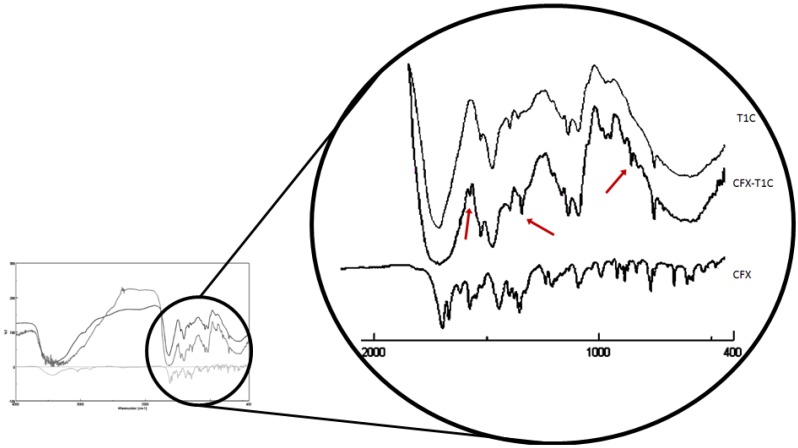
FT-IR spectra of T1C, CFX-T1C and CFX.

Figure 3 shows the FT-IR spectra of collagen, CFX-T1C conjugate and ciprofloxacin. In particular, the FT-IR spectrum of CFX-T1C conjugate shows three different bands in the fingerprint zone ascribable to the presence of the drug in the polymeric chain. Although it is difficult to pick out individual bonds in this region, we can give the following assignments: The band at about 1,620 cm^−1^ is ascribable to the quinoline rings, and the band at 1,410 cm^−1^ to the carbonyl group of ciprofloxacin.

**Figure 4:**
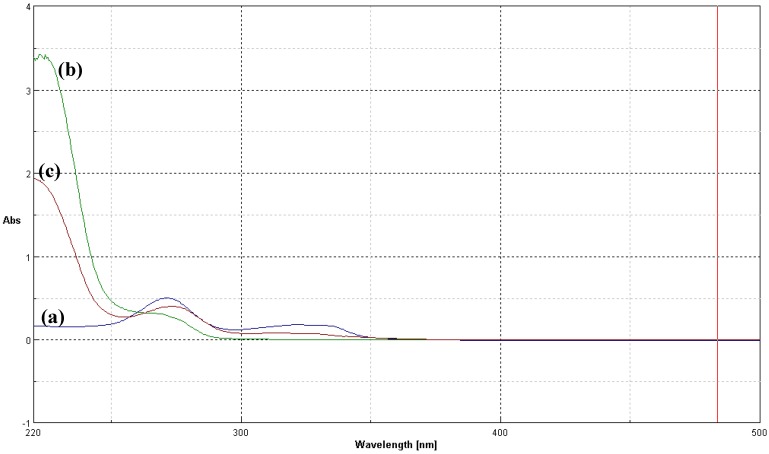
UV-VIS spectra of CFX (**a**); collagen (**b**) and CFX-T1C conjugate (**c**).

A further confirmation of CFX incorporation was obtained by comparing the UV absorption spectra of collagen and CFX-T1C conjugate (Figure 4). In the CFX-T1C UV spectrum, indeed, the absorption is shifted to higher wavelengths as a consequence of the extension of the conjugation due to the formation of covalent bonds between collagen reactive groups and the CFX molecule.

### 3.2. *In Vitro* Wound Healing Assays

#### 3.2.1. Antibacterial Assay

Wound healing is a complex series of biochemical and cellular events. Wounds provide an environment for the growth of microorganisms and an infected wound is less likely to heal, thus, in order to promote rapid and effective wound healing, the removal and the prevention of further infection could be relevant. 

In the aim to evaluate the antibacterial activity, different concentrations of the synthesized polymeric conjugate were tested against *S. aureus* and *E. coli*. These microorganisms were selected for this study as test organisms due to the frequency with which they infect wounds [13,14]. 

CFX-T1C conjugate was found to be active against both *S. aureus* and *E. coli *and the obtained MIC values were 12 µg/mL and 0.8 µg/mL, respectively. The obtained data confirmed that the antimicrobial activity of ciprofloxacin was not lost after the covalent incorporation of the drug into the polymeric chain or following exposure to the adopted reaction conditions. In particular, among the used test organisms, *E. coli* was found to be more sensitive than *S. aureus*.

The antibacterial test was repeated employing water/TSB mixture and water/TSB mixture inoculated with each test bacterium in the microtiter plates as negative and positive controls, respectively. The antibacterial activity results for the negative and positive controls were also observed as expected. There was no growth of bacteria in the negative controls; whereas both bacteria grew well in the positive controls.

As reported in our previous work [9,11,12], the adopting grafting procedure leads to the formation of C–C covalent bonds between the biopolymer and the drug, which are not hydrolysable by enzymes. In this study, no experimental data regarding the enzymatic stability of the synthesized ciprofloxacin-collagen conjugate (CFX-T1C) were collected, but from the literature it is known that collagenase is the only enzyme able to cleave collagen triple helical regions under physiological pH and temperature conditions [15]. Based on these considerations, it is reasonable to hypothesize that the polymeric material could serve both as an intact conjugate with antimicrobial properties and, after the matrix degradation, as collagen segments with a lower molecular weight.

#### 3.2.2. Influence of Catechin Blended CFX-T1C on Human ﬁbroblast Skin Cells *in Vitro*

Skin fibroblast proliferation is important in tissue repair as fibroblasts are involved in migration, proliferations, contractions and collagen production [16,17]. The ability to stimulate fibroblast cell growth is a useful model for testing wound healing activity *in vitro *[18,19]. 

There are several assays for the evaluation of proliferation and cytotoxic effects of chemicals on cultured cells, and the neutral red assay represents one of most representative of these techniques. Although the Alamar assay has the advantage of being more sensitive than colorimetric systems, the neutral red assay is widely reported in the literature to evaluate cell viability [20,21,22,23]. Moreover, many studies report the use of this assay with the aim to examine cytotoxic effects and viability of cells, including fibroblasts, exposed to ciprofloxacin [24,25,26,27]. Thus, in this study the neutral red assay was chosen to evaluate the fibroblast cell growth in the presence of the synthesized conjugate and in the presence of catechin blended conjugate. The obtained results indicate that the effects on fibroblast proliferation are dose-dependent confirming the efficiency of the performed assay.

In the literature, many studies have highlighted the involvement of flavonoid compounds in wound healing processes by stimulating fibroblast growth [28,29].

**Figure 5:**
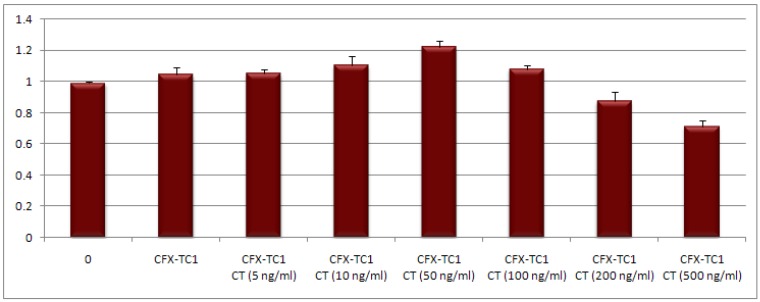
Influence of catechin blended CFX-T1C on human dermal skin fibroblasts *in vitro*.

The obtained findings are reported in Figure 5 and indicate that the use of catechin blended CFX-T1C may promote wound healing by the stimulation of fibroblasts. Furthermore, the obtained results suggest that the effects on the fibroblast proliferation are dose-dependent. 

Stimulation of fibroblast cell growth was observed at catechin concentrations in the range of 5–100 ng/mL. In particular, a higher improvement of cell proliferation occurs for a CT concentration of 50 ng/mL. At higher concentrations (200–500 ng/mL) there is evidence of cytotoxicity or inhibition since the absorbance was less than in the control. 

The data indicate that also the polymeric conjugate, without CT addition, is able to influence fibroblast proliferation due to the intrinsic antibacterial activity of the biomaterial that preserves cells. 

## 4. Conclusions

In this work, a fluoroquinolone-polymer conjugate to be applied as new functional biomaterial in wound healing was synthesized and characterized in terms of antibacterial activity. 

Ciprofloxacin was covalently incorporated into collagen chains using a free-radical grafting procedure carried out in aqueous media and involving the use of an ascorbic acid/H_2_O_2 _redox pair as biocompatible initiator system. The mild reaction conditions, together with the absence of toxic reaction products and the high yields, make the adopted synthetic procedure very useful to prepare polymeric conjugates characterized by antibacterial properties. 

The covalent insertion of CFX in the polymeric chain was confirmed by performing FT-IR and UV analyses. The antibacterial properties of the synthesized CFX-T1C conjugate were demonstrated by specific assay employing *S. aureus* and *E. coli* as test organisms, due to the frequency with which they infect wounds. The *in vitro* ability of CFX-T1C, in absence and in presence of a flavonoid compounds such as catechin, to stimulate ﬁbroblast growth was also observed. 

The obtained findings support the idea that the synthetic strategy allows improving the properties of a natural polymer, such as collagen, and that CFX-T1C conjugate represents an effective antibacterial device showing the ability to influence fibroblast proliferation. Based on these considerations, the synthesized conjugate could be applied as a new functional biomaterial in wound treatment encouraging the healing process. 
